# Novel SiC Trench MOSFET with Improved Third-Quadrant Performance and Switching Speed

**DOI:** 10.3390/mi15020254

**Published:** 2024-02-08

**Authors:** Yangjie Ou, Zhong Lan, Xiarong Hu, Dong Liu

**Affiliations:** 1 School of Electrical Engineering, Southwest Jiaotong University, Chengdu 611756, China; oyj194116@my.swjtu.edn.cn (Y.O.); lanzhong@swjtu.edu.cn (Z.L.); 2 School of Science, Xihua University, Chengdu 610039, China; hxr2013@mail.xhu.edu.cn

**Keywords:** SiC MOSFET, low-barrier diode, L-shape gate-source, bipolar degradation, gate–drain capacitance

## Abstract

A SiC double-trench MOSFET embedded with a lower-barrier diode and an L-shaped gate-source in the gate trench, showing improved reverse conduction and an improved switching performance, was proposed and studied with 2-D simulations. Compared with a double-trench MOSFET (DT-MOS) and a DT-MOS with a channel-MOS diode (DTC-MOS), the proposed MOS showed a lower voltage drop (*V*_F_) at *I*_S_ = 100 A/cm^2^, which can prevent bipolar degradation at the same blocking voltage (BV) and decrease the maximum oxide electric field (*E*_mox_). Additionally, the gate–drain capacitance (*C*_gd_) and gate–drain charge (*Q*_gd_) of the proposed MOSFET decreased significantly because the source extended to the bottom of the gate, and the overlap between the gate electrode and drain electrode decreased. Although the proposed MOS had a greater *R*_on,sp_ than the DT-MOS and DTC-MOS, it had a lower switching loss and greater advantages for high-frequency applications.

## 1. Introduction

Nowadays, silicon carbon (SiC) is widely used in many applications because of its high critical electric field and superior thermal conductivity [1,2]. The SiC MOSFET has a lower on-resistance and a faster switch speed compared with the Si-insulated Gate Bipolar Translator (IGBT) [3,4]. However, the body diode of the SiC MOSFET has a high on-state voltage drop of about 2–3 V because of its wide bandgap. Additionally, when the body diode operates in bipolar mode, basal plane dislocations (BPDs) and stacking faults (SFs) are generated because of the recombination energy of the electrons and holes, and these faults cover most of the junction area and cause conduction losses to increase [5,6,7,8]. Thus, the SiC MOSFET usually reverse-parallels a freewheeling diode to suppress the body diode; this extra diode not only increases the package size but also increases the parasitic inductance, which limits the switching frequency of the MOSFET [9,10].

One possible way of solving this problem is to integrate a unipolar diode into the MOSFET cell—in particular, a Schottky Barrier Diode (SBD)/Junction Barrier Controlled Schottky Diode (JBS) [9,11,12,13,14,15]. A disadvantage of these integrated unipolar diodes is the increased leakage current in the blocking state for the MOSFET [16]. The use of a built-in channel diode is another option that can improve the reverse-recovery characteristics of the MOSFET, showing better switching characteristics, but the reliability problem caused by thin gate oxide still needs further research [17,18]. In recent research, low-barrier diodes (LBDs) have been adopted for their enhanced third-quadrant and switching performance in planar MOSFETs [19], but the planar structure limits the MOSFET’s usage in high-power applications because of its wide cell pitch and its high specificity of resistance (*R*_on,sp_).

This paper proposes a 1200 V L-shaped split-gate trench SiC MOSFET integrated with a low-barrier diode. This structure can inhibit the reverse conduction of the body diode to avoid the effects of bipolar degradation and to extend the source to the bottom of the gate, forming a split gate to reduce the *C*_rss_ [20,21,22] and to achieve a fast switching speed. This study was carried out with numerical TCAD, and some essential models were included such as the Fermi–Dirac, incomplete-ionization, Shockley–Reed–Hall and Auger combination, Lombardi (CVT), impact-ionization, and band-narrowing models [23,24,25]. A channel mobility of 50 cm^2^/(Vs) was used [26]. The structure achieved a lower *V*_F_, *C*_gd_, and *Q*_gd_ and lower switching losses compared with a DT-MOS [27] and a DT-MOS with an MOS-channel diode (DTC-MOS) [18], and it also reduced the maximum oxide electric field (*E*_mox_).

## 2. Device Structure and Mechanism

**Figure 1:**
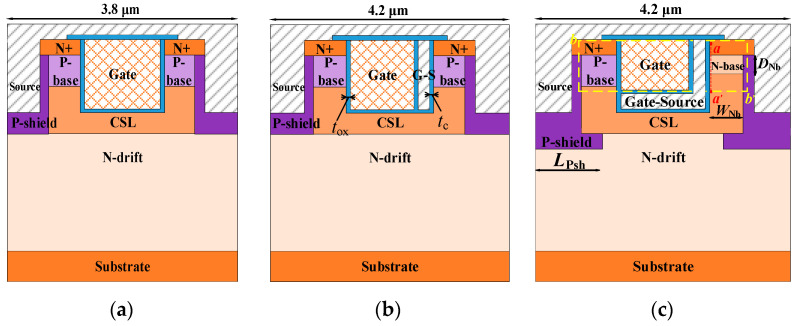
Schematic cross-sectional structures of the (**a**) DT-MOS, (**b**) DTC-MOS, and (**c**) proposed MOSFET.

Figure 1 shows the schematic structures of the (a) DT-MOS, (b) DTC-MOS, and (c) proposed MOS. Based on the DTC-MOS, the proposed MOS turns part of the polysilicon gate to the source and extends to the bottom of the gate, forming an “L-shape” split gate. The gate-source connects to the source, so the overlap between the gate and drain decreases, which leads to a decrease in the *C*_gd_. Meanwhile, at the right half-cell, the p-base turns into an n-base, so a low-barrier diode is integrated into this structure to improve its reverse conduction. The P-shield extends to the current spreading layer (CSL), and it decreases the *E*_mox_ in the blocking state and increases the BV, improving the device’s reliability.

This device is based on a 4H-SiC, with the doping concentration and thickness of the N-drift set at 8 × 10^15^ cm^−3^ and 9 μm, respectively. The P-base region in all the devices had a doping concentration of 2 × 10^17^ cm^−3^ and a thickness of 0.5 μm. The N-base had a doping concentration of 3 × 10^16^ cm^−3^ and a thickness of 0.3 μm. The P-shield had a doping concentration of 2 × 10^18^ cm^−3^ and a thickness of 0.3 μm. The CSL had a doping concentration of 8 × 10^16^ cm^−3^ and a depth of 1.7 μm. The depth of the source trench and gate trench was 1.4 μm for both devices. The thickness of the gate oxide was 50 nm for both the N-base and P-base to improve the device reliability in the DT-MOS and proposed MOS. Considering the sufficient volume of the gate and the electric field, the distance of the oxide between the gate and gate-source and the thickness of the gate-source was 0.1 μm for the DTC-MOS and the proposed MOS. The cell pitch was 3.8 μm for the DT-MOS, and that of the other two devices was 4.2 μm. The main structure parameters of the DT-MOS, DTC-MOS, and proposed MOS are shown in Table 1.

**Figure 2:**
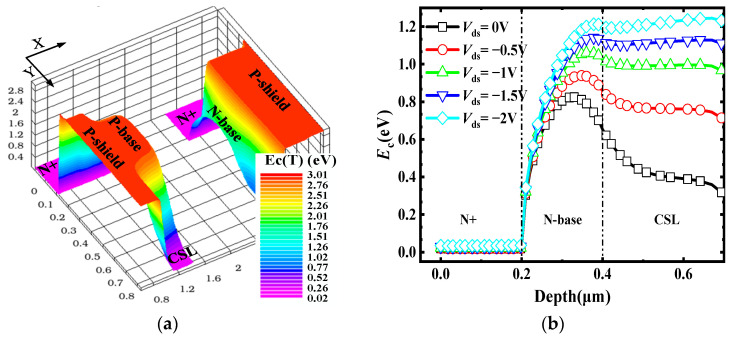
(**a**) Three-dimensional *E*_C_ distribution of the proposed MOSFET at zero bias (b–b’—the yellow dashed line in Figure 1). (**b**) *E*_C_ distribution of the SiO_2_/SiC interface (a–a’—the red dashed line in Figure 1) at different *V*_ds_.

Figure 2a shows the three-dimensional conduction band energy (*E*_C_) distribution of the 4H-SiC in the proposed MOS structure at zero bias. The *E*_C_ decreased from the P-shield to the N-base at the right half-cell. The high doping of the P-shield and the low doping of the N-base led to a rapid depletion of the N-base region at zero bias, preventing the formation of a conducting channel. Therefore, there was no impact on the *BV* at low doping concentrations. At zero bias, the *E*_C_ of the N-base was lower than the P-base, allowing electrons to overcome the potential barrier at a low *V*_ds_. Figure 2b shows the *E*_C_ distribution along the a–a’ line (shown in Figure 1) at different *V*_ds_. As *V*_ds_ decreased, the *E*_C_ increased in both the N-base and CSL. However, the *E*_C_ of the CSL increased faster than the N-base region. At *V*_ds_ = −1 V, the potential barrier became very low, allowing electrons to overcome the potential barrier, turning on the low barrier diode.

The potential barrier model for LBD in a planar MOSFET is given by [19]:

(1) $$V_{\mathrm{bi},\mathrm{LBD}}=\frac{\phi_{\mathrm{Si}\_\mathrm{SiC}}-\frac{qN_{\mathrm{Nb}}}{2\varepsilon_{\mathrm{SiC}}}W_{\mathrm{Nb}}^{2}}{\frac{\varepsilon_{\mathrm{ox}}W_{\mathrm{Nb}}}{\varepsilon_{\mathrm{SiC}}t_{\mathrm{ox}}}+1}+\chi_{\mathrm{Si}\_\mathrm{SiC}}$$

where $\phi_{Si\_SiC}$ and $\chi_{Si\_SiC}$ are the work function and electron affinity difference between the Si and SiC. $\varepsilon_{OX}$ and $\varepsilon_{SiC}$ are the permittivity of the SiO_2_ and SiC. *N*_Nb_ is the doping concentration of the N-base. The *W*_Nb_ is the width of the N-base. The structure of the low barrier diode in a planar structure and trench structure is the same, being formed by the N+ polysilicon, oxide, the low doping concentration N region, and the high doping concentration P region. Although the formula was obtained for planar structures [19,28], it is also applicable to trench structures. The t_c_ is the thickness of the oxide between the gate-source and the N-base, which is fixed at 50 nm in the proposed MOS, as it has a great influence on device reliability. The t_c_ of the DTC-MOS was 20 nm, to easily turn on the built-in diode. From equation (1), *N*_Nb_ and *W*_Nb_ had an impact on the potential barrier of the LBD. Additionally, the thickness of the N-base (*T*_Nb_) and the length of the P-shield (*L*_Psh_) also influenced the resistance of the LBD, which in turn affects the *V*_F_. Therefore, these parameters were optimized.

## 3. Simulation Results and Analysis

**Figure 3:**
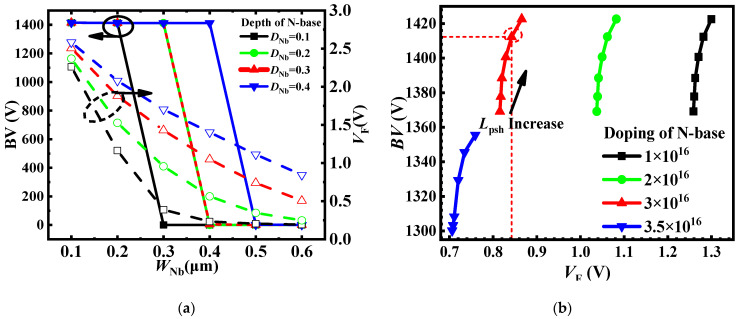
(**a**) Influence of *D*_Nb_ and *W*_Nb_ on BV and *V*_F_; (**b**) *BV*, *V*_F_ at different *L*_Psh_ and *N*_Nb_.

Figure 3a illustrates the impact of *W*_Nb_ and *D*_Nb_ on the *BV* and *V*_F_. The solid circles represent *BV* values and the dashed circles represent *V*_F_ values. The *W*_Nb_ varied from 0.1 μm to 0.6 μm in steps of 0.1 μm. As *W*_Nb_ increased, the reverse current path expanded, which led to a decrease in *V*_F_. However, the breakdown turned into a punch-through breakdown, which made the device unable to withstand high voltages. With increasing *D*_Nb_, the length of the potential barrier increased, leading to an increase in *V*_F_. In this case, a longer *W*_Nb_ was required to trigger a punch-through breakdown. It is worth noting that when *D*_Nb_ exceeded 0.3 μm, the leakage current of the proposed MOS became comparable to the DT-MOS, which will be further discussed later. To tradeoff the *BV*, *V*_F_, and leakage current, the optimal values of *W*_Nb_ = 0.3 μm and *D*_Nb_ = 0.3 μm were selected.

Figure 3b shows the tradeoff between the *BV* and *V*_F_ for the proposed MOS, considering different values of *L*_Psh_ and *N*_Nb_. As the *L*_Psh_ increased from 1 μm to 1.5 μm in steps of 0.1 μm, the depletion region extended, leading to a decrease in *E*_mox_ and an increase in *BV*. However, the current path became narrow, leading to an increase in *R*_on,sp_ and *V*_F_ because of the change in the JFET resistance. With increasing *N*_Nb_, the potential barrier of the low barrier diode reduced, leading to a decrease in *V*_F_. However, with high doping of *N*_Nb_, the *BV* dropped below 1400 V, as shown for *N*_Nb_ = 3.5 × 10^16^ cm^−3^. At low *N*_N_ values_,_ the breakdown point occurred at the P-shield/N-drift junction, so the *BV* did not change with different *N*_Nb_ values. The change in *N*_Nb_ had no influence on *R*_on,sp_. However, with increasing *L*_Psh_, the *R*_on,sp_ increased from 1.84 mΩ × cm^−2^ to 4.58 mΩ × cm^−2^. In order to tradeoff *BV*, *V*_F_, and *R*_on,sp_, *L*_Psh_ = 1.3 μm and *N*_Nb_ = 3 × 10^16^ cm^−3^ were selected, represented by the red circle in Figure 3b.

**Figure 4:**
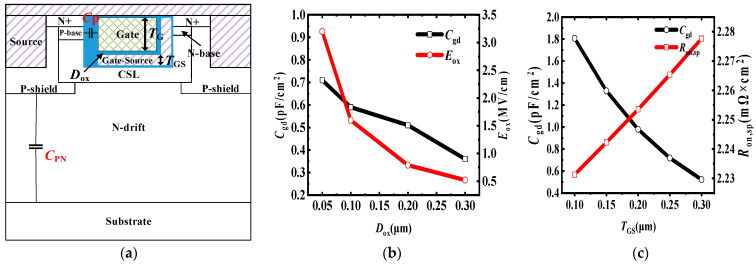
(**a**) Parasitic capacitance for the *C*_gd_ of the proposed MOS. (**b**) Influence of device characteristics on the distance between the gate and gate-source (**c**) Influence of *C*_gd_ and *R*_on,sp_ on the thickness of the gate-source.

The main parameters of the gate trench are shown in Figure 4a. The thickness of the gate trench was fixed at 1.4 μm. Figure 4b shows the influence of the distance between the gate and the gate-source (*D*_ox_) on the *C*_gd_ and oxide electric field (*E*_ox_). The voltage between the gate and the gate-source was set to 15 V. When *D*_ox_ was 0.1 μm for both the bottom and side wall of the gate, the *E*_ox_ was 1.5 MV/cm, which corresponds to the simulation results. The thickness of the gate-source (*T*_GS_) was fixed at 0.1 μm, and the thickness of gate poly (*T*_G_) changed as *D*_ox_ increased or decreased. With increasing *D*_ox_, the *BV* and *V*_F_ had no influence, so they are not included in Figure 4b. The *D*_ox_ has little influence on *C*_gd_. Therefore, when *D*_ox_ was greater than 0.1 μm, *E*_ox_ was already less than 3 MV/cm. In order to facilitate subsequent simulations and ensure a sufficient volume of gate poly for adjusting the gate resistance, *D*_ox_ = 0.1 μm was selected.

The influence of the device characteristics on *T*_GS_ is shown in Figure 4c; the *D*_ox_ was fixed at 0.1 μm. With increasing *T*_GS_, there was no influence on *BV* and *V*_F_, which is not shown in the figure. *R*_on,sp_ increased from 2.23 mΩ∙cm^2^ to 2.28 mΩ∙cm^2^, because the CSL, oxide, and gate poly formed an MIS structure, which increased the electron concentration of the CSL during conduction; this effect weakened as *T*_G_ decreased. 

With increasing *T*_GS_, *C*_gd_ decreases; this is because the gate-source extends to the bottom of the gate poly, resulting in a significant decrease in the overlap between the gate electrode and drain electrode. In this case, the *C*_gd_ can be expressed as:

(2) $$C_{\mathrm{gd}}=\frac{C_{p}\times C_{\mathrm{PN}}}{C_{p}+C_{\mathrm{PN}}}$$

As shown in Figure 4a, *C*_p_ is the oxide capacitance between the P-base and gate electrode, which is related to the thickness of the oxide and the overlap between the gate poly and the P-base. *C*_PN_ is the junction capacitance, which is completely independent of the gate parameters, and the *C*_PN_ decreases as *V*_ds_ increases. When increasing *T*_GS_ or *D*_ox_, the *T*_G_ decreases, resulting in a decreased overlap between the gate poly and the P-base, thus causing a decrease in *C*_p_. Meanwhile, the *T*_G_ has no influence on *C*_PN_, so the *C*_gd_ will decrease. However, it is worth noting that the *C*_gd_ is already sufficiently small, and further decreasing *C*_p_ cannot significantly change the *C*_gd_. To ensure a suitable gate resistance for device, a sufficient volume of gate poly must be considered, which cannot be reflected in a simulation. Therefore, *T*_GS_ = 0.1 μm was selected for further simulations. According to Figure 4b,c, the internal parameters of the gate trench have little influence on the performance of the device when the resistance of the gate poly is not considered; this shows that the proposed MOS has a wide process window for forming the L-shape gate-source.

**Figure 5:**
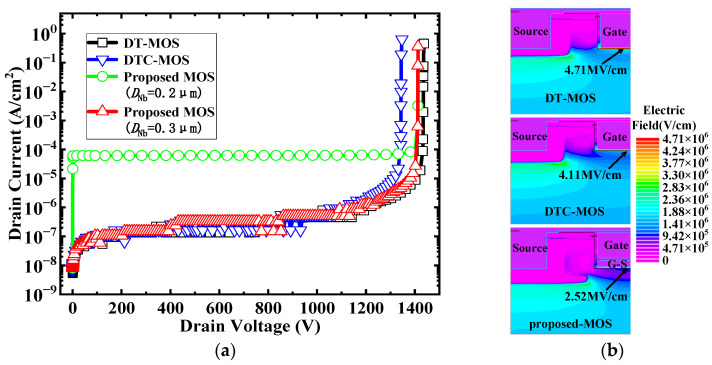
(**a**) BV characteristics (**b**) electric field distributions with a drain bias at 1200 V of the DT-MOSF, DTC-MOS, and proposed MOS.

Figure 5a shows the leakage current and blocking voltage for the three devices. The DT-MOS and proposed MOS blocking voltage exceeded 1400 V. However, the BV of the DTC-MOS was only 1340 V. This indicates that a wide cell pitch results in a decrease in the BV, while the extended P+ shield helps to improve the BV. For the proposed MOS, the leakage current increased faster at *D*_Nb_ = 0.2 μm. However, when *D*_Nb_ = 0.3 μm, the BV was the same as *D*_Nb_ = 0.2 μm, the leakage current decreased to the level of the DT-MOS. This is because the leakage current is related to the parameters of the N-base before breakdown and the blocking voltage is related to the P-shield/N-drift junction, where the electric field is highest in the SiC region and avalanche breakdown occurs. The electric field distribution of the three devices at 1200 V is shown in Figure 5b. The *E*_mox_ was located at the bottom of the oxide for all the devices. Compared with the DT-MOS and DTC-MOS, the proposed MOS had an extended P-shield, which was able to expand the depletion layer and provide better protection effects to the oxide. As a result, the *E*_mox_ was only 2.52 MV/cm, while the *E*_mox_ of the other devices was higher than 4 MV/cm. With a high *E*_mox_, a Fowler–Nordheim tunneling current may be generated; this carries electrons through the oxide layer, breaking the Si-O bond over time and generating defects, leading to a full breakdown of the SiO_2_ layer [29], which has a great influence on device reliability.

**Figure 6:**
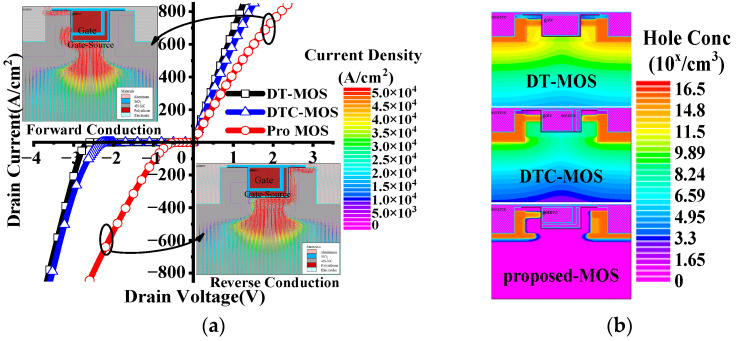
(**a**) I–V characteristics of the three devices; (**b**) hole concentration distributions at *I*_s_ = 100 A/cm^2^.

The I–V characteristic is shown in Figure 6a. In forward conduction, the *R*_on,sp_ of the DT-MOS and DTC-MOS was smaller than for the proposed MOS; this is because the DT-MOS has two channel paths for conduction, and because both DT-MOS and DTC-MOS do not extend the P-shield, which increases JFET resistance. With a low barrier diode, the *V*_F_ of the proposed MOS decreased significantly. The *V*_F_ was 2.85 V, 2.63 V, and 0.85 V at 100 A/cm^2^ for the DT-MOS, DTC-MOS, and proposed MOS, respectively. The current vector of the forward and reverse conduction is also shown in Figure 6a. It can be seen that there was only one current path for both conduction conditions. The current flows from the N+ region through the N-base to the drift region in reverse conduction, while the current flows from the drift region through the P-base to the N+ region in forward conduction. Figure 6b shows the hole concentration at *I*_s_ = 100 A/cm^2^ of all the devices. In reverse conduction, the drift region of the DT-MOS obtained a high concentration of holes, which causes bipolar degradation [5].

**Figure 7:**
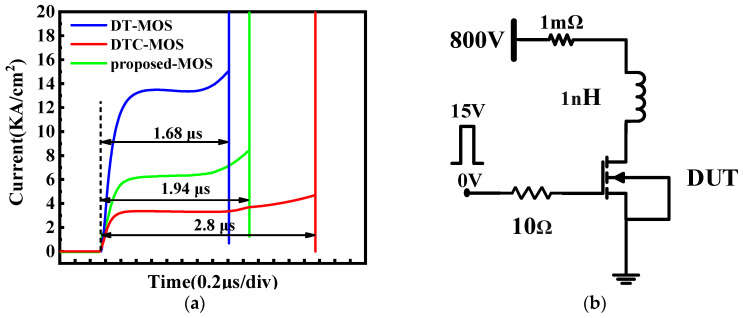
(**a**) Short circuit characteristics of the three devices; (**b**) short-circuit test circuit.

The short-circuit (SC) test results for the DT-MOS, DTC-MOS, and proposed MOS are shown in Figure 7. The SC test circuit used in the simulation is shown in Figure 7b. The bus voltage was 800 V. The stray inductance and resistance was 1 nH and 1 mΩ, respectively. The gate resistance was 10 Ω. A single pulse of 0 V/15 V gate bias was applied to the gate contact until the device failed due to thermal runaway caused by excessive temperatures. The time from device turn-on to failure was 1.6 μs, 1.9 μs, and 2.8 μs for the DT-MOS, DTC-MOS, and proposed MOS, respectively. For the DT-MOS, the highest saturation current caused a faster temperature rise, leading to earlier device failure. Due to the single current channel and depletion layer extension of the P-shield region, the proposed MOS exhibited the lowest saturation current. As a result, the proposed MOS achieved the longest time until failure.

**Figure 8:**
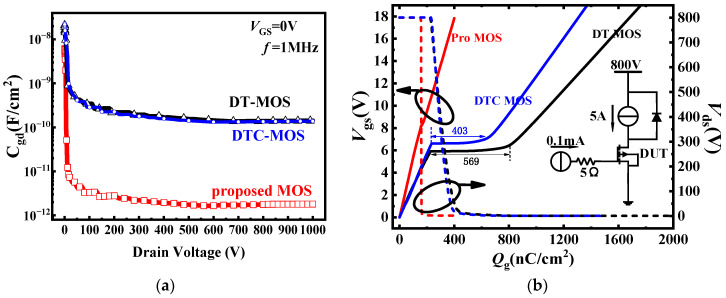
(**a**) C–V characteristics; (**b**) Gate Charge of three devices.

In the proposed MOS, the gate-source extended to the bottom of the gate, leading to a decrease in the overlap between the gate and the drain. As a result, the proposed MOS had the lowest *C*_gd_ compared to the DT-MOS and DTC-MOS, as shown in Figure 8a. While switching transients, the time constant is determined by the junction capacitance and gate resistors, which impact the switching speed of the devices. With a smaller capacitance, the devices switch at a faster speed. The *C*_gd_ was 141.68 pF/cm^2^, 136 pF/cm^2^, and 1.81 pF/cm^2^ for the DT-MOS, DTC-MOS, and proposed MOS, respectively. Figure 8b shows the gate charge for the three devices; the *Q*_gd_ of the DT-MOS was 569 nC/cm^2^ and the *Q*_g_ (*V*_gs_ = 15 V) was 1467 nC/cm^2^. The *Q*_gd_ of the DTC-MOS was 406 nC/cm^2^ and the *Q*_g_ was 1136 nC/cm^2^. However, the *Q*_gd_ of the LST-MOSFET was 6.7 nC/cm^2^ and the *Q*_g_ was 333 nC/cm^2^; this result is consistent with the results for the *C*_gd_, indicating that the proposed MOS can significantly reduce switching losses.

**Figure 9:**
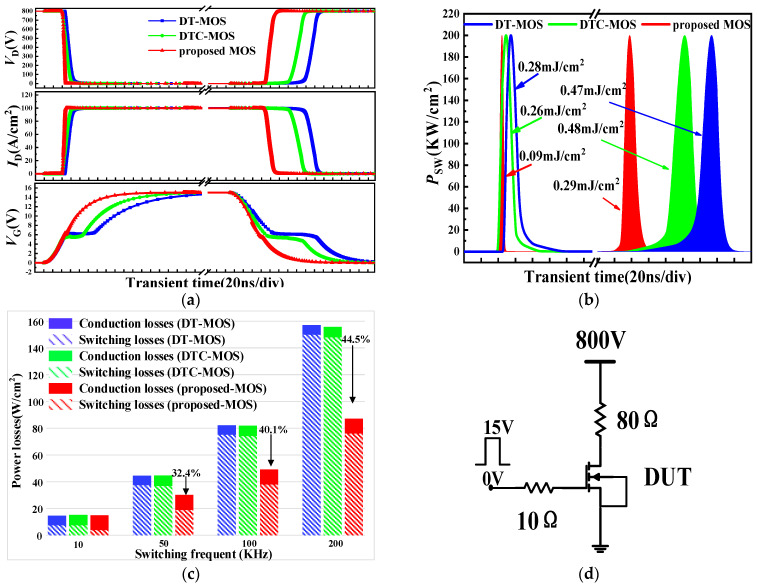
The switching waveforms of the DT-MOS, DTC-MOS, and Proposed MOS, respectively; (**a**) Turn-on and turn-off waveforms; (**b**) Turn-on loss and turn-off loss waveforms; (**c**) total power losses as a function of switching frequency *f*; (**d**) resistance switching circuit for simulations.

The switching waveforms and test circuit of the three devices are shown in Figure 9. Figure 9d shows the resistance switch circuit used in the simulation, with a load current of 10 A (100 A/cm^2^) at a normal current density. As can be seen in Figure 9a, the proposed MOS exhibited a lower *Q*_gd_ compared to the other devices. The miller plateau almost disappeared, leading to a faster transition of *V*_gs_, which is consistent with the *C*_gd_ results. This characteristic resulted in a significantly faster switching speed for the proposed MOS compared to the other devices, thereby reducing switching losses. From Figure 9b, the turn-on loss (*E*_on_) and turn-off loss (*E*_off_) of the DT-MOS was 0.28 mJ/cm^2^ and 0.47 mJ/cm^2^ and for the DTC-MOS was 0.26 mJ/cm^2^ and 0.48 mJ/cm^2^. However, for the proposed MOS, the *E*_on_ and *E*_off_ decreased to 0.09 mJ/cm^2^ and 0.29 mJ/cm^2^. The switching losses (*E*_SW_) consisted of the *E*_on_ and *E*_off_; the *E*_SW_ of the proposed MOS was 49.3% and 48.6% lower than that of the DT-MOS and DTC-MOS, respectively.

The total power losses (*P*_t_) consist of conduction power losses and switching losses. When the device is operating under a square wave with a period T and a duty cycle D, the *P*_t_ can be expressed as (3):

(3) $$P_{t}=V_{d}\times I_{d}\times D+E_{SW}\times f$$

When the device operated at 100 A/cm^2^, the *V*_ds_ was 0.132 V, 0.156 V, and 0.223 V for the DT-MOS, DTC-MOS, and proposed MOS, which is consistent with the *R*_on,sp_. The switching frequency, *f*, is related to the period, T, by the formula f=1T. Although the *R*_on,sp_ of the proposed MOS was greater than that of the DT-MOS and DTC-MOS, the switching losses were the main contributor to power loss at high frequencies. Working at high frequencies can effectively reduce the total power losses of the device; it is worth it to increase the on-resistance slightly to achieve smaller switching losses at high frequencies. Figure 9c shows the total power loss as a function of *f* for the three devices, when a D of 50% was assumed. When the switching frequency was 50 KHz, the proposed MOS achieved the lowest power loss compared to the other devices due to its lower switching loss. At a switching frequency of 200 KHz, the *P*_t_ of the proposed MOS was 44.5% lower than the DT-MOS. With increasing *f*, the deference in *P*_t_ between the DT-MOS and the proposed MOS gradually increased.

**Figure 10:**
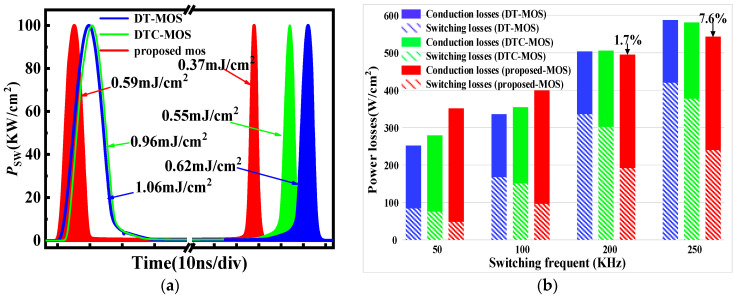
Device works at 500 A/cm^2^; (**a**) Turn-on loss and turn-off loss waveforms; (**b**) total power losses as a function of switching frequency *f*.

The switching condition at a high current density (500 A/cm^2^) is shown in Figure 10. As the current density increased, both conduction losses and switching losses increased significantly. The *E*_SW_ of the three devices is shown in Figure 10a. The proposed MOS value was 0.96 mJ/cm^2^, which was 42.9% and 36.4% lower than the DT-MOS and DTC-MOS. However, the conduction losses of the proposed MOS increased faster than the other devices at 500 A/cm^2^. As a result, the *P*_t_ of the proposed MOS was the highest before *f* = 200 KHz, as shown in Figure 10b. At a *f* of 250 KHz, the *P*_t_ of the proposed MOS was only 7.6% lower than that of the DT-MOS. Comparing the work conditions between a normal current density and a high current density, it is more favorable for the proposed MOS to work at a normal current density.

However, high switching speeds and frequencies may present a greater switching oscillation challenge [30]. By adding RC snubbers [31], reducing the switching speed [32], or using active gate control techniques [33], the switching oscillation will be suppressed. However, the mentioned methods for suppressing switching oscillation will lead to an undesirable increase in switching time and switching losses [30]. There is a tradeoff between power losses and switching oscillation. An electronic structure that has transient part-time symmetry triggered by the switching-on and off of electronic devices can release oscillation energy, while still maintaining the very low loss state [34]. This may be a good choice for suppressing switching oscillation in the future. The comprehensive performance of the three devices is shown in Table 2.

**Figure 11:**
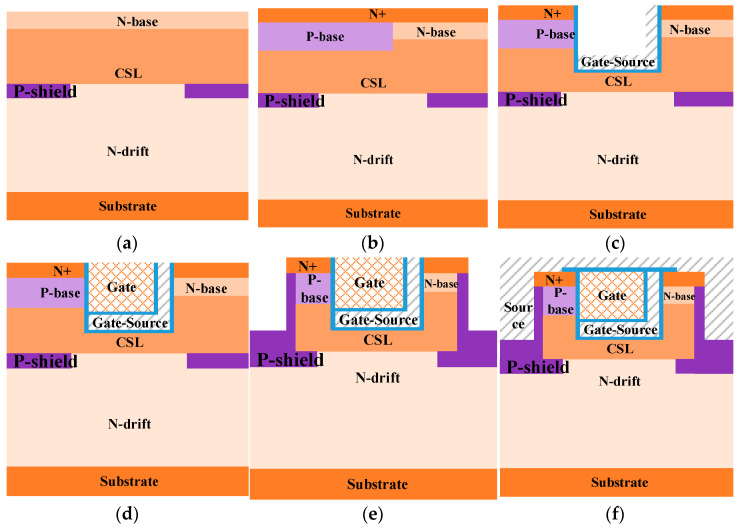
Key fabrication process flows for the proposed MOSFET: (**a**) Form P-shield layer. (**b**) Form P-base layer and N-source layer. (**c**) Etch to form gate trench and form oxide by CVD to form the gate oxide; deposit and etch polysilicon to form gate-source. (**d**) Deposit oxide and polysilicon to form gate. (**e**) Etch to form source trench and ion implantation to form the P+. (**f**) Form source electrode.

Figure 11 shows a possible process for the proposed MOS. The process starts with the formation of the P-shield region after epitaxy, as shown in Figure 11a. Then, the P-base and N-source are formed by ion implantation followed by high-temperature annealing, as shown in Figure 11b. After this, the gate trench is etched, the gate oxide is formed by chemical vapor deposition (CVD), and the polysilicon is deposited and etched to form the gate-source, which is shown in Figure 11c. Then, the oxide is deposited and the N+ polysilicon is deposited and etched to form the gate electrode, which is shown in Figure 11d. Figure 11e shows the etching of the source trench and tilted implantation to form the P+ region along the sidewall of the source trench. Finally, Figure 11f shows the deposition of a passivation layer, the etching of the contact window, and the formation of the ohmic contact.

## 4. Conclusions

In this paper, a SiC novel MOSFET is proposed and studied by TCAD simulations. The proposed MOS integrates a low barrier diode and has a gate-source structure located under the bottom of gate. The simulation results demonstrate that the proposed MOSFET has a smaller *V*_F_ compared to the DT-MOS and DTC-MOS because of the low barrier diode, which suppresses the conduction of the body diode. This allows the proposed MOS to operate under unipolar operations with reverse conduction, preventing the effects of bipolar degradation. The influence of the main parameters of LBD on device performance has been studied, and the optimal value has been determined. Additionally, the length of the P-shield has been studied to achieve a low *E*_mox_ and high blocking voltage. The parameters of the gate trench have also been studied, which show a high process tolerance for forming an L-shape without affecting the static performance.

In addition to the static performance, the *C*_gd_ and *Q*_gd_ of the three devices were compared. Due to a reduction in the overlap between the gate electrode and drain electrode, the proposed MOS achieved the lowest *C*_gd_ and *Q*_gd_. As a result, the proposed MOSFET is able to achieve better switching speeds and lower switching losses. The proposed MOS achieved the lowest total power losses under 50 KHz and higher switching frequencies with a normal current density. This indicates that the proposed MOSFET has more advantages in high frequency switching applications.

## Figures and Tables

**Table 1:** Structural parameters of the three devices.

Symbol	Description	DT-MOS	DTC-MOS	Proposed MOS
W_cell_	Width of cell pitch, μm	3.8	4.2	4.2
t_ox_	Thickness of gate oxide, nm	50	50	50
t_c_	Thickness of gate oxide, nm	50	20	50
W_ST_	Width of source trench, μm	0.6	0.7	0.7
W_GT_	Width of gate trench, μm	1	1.2	1.2
T_Nd_	Thickness of N-drift, μm	9	9	9
N_Nd_	Concentration of N-drift, cm^−3^	8 × 10^15^	8 × 10^15^	8 × 10^15^

**Table 2:** Comparison of the three devices’ characteristics.

Symbol	DT-MOS	DTC-MOS	Proposed MOS
*BV* [V]	1434	1343	1411
*E*_mox_ [V cm^−1^]	4.71	4.11	2.52
*V*_F_ [V]	2.86	2.63	0.85
*R*_on,sp_ [mΩ∙cm^2^]	1.42	1.56	2.23
*Q*_gd_ [nC/cm^2^]	569	406	6.7
*E*_on_ [mJ/cm^2^]	0.28	0.26	0.09
*E*_off_ [mJ/cm^2^]	0.47	0.48	0.29

## Data Availability

Data are contained within the article.
